# Role of Motivation in the Progression of Problem Gambling: A Comparison of Early and Late Adults

**DOI:** 10.1007/s10899-024-10331-5

**Published:** 2024-06-22

**Authors:** Yasunobu Komoto

**Affiliations:** Department of Psychiatry, Yoshino Hospital, 2252 Zushimachi, Machida city, Tokyo 1940203 Japan

**Keywords:** Gambling, Motivation, Social recognition, Amotivation

## Abstract

Motivation plays a dominant role in gambling progression. Most studies using motivational scales have revealed that certain motivations are associated with problem gambling. However, age differences were found to be negligible in gambling motivation. This study aimed to examine the role of motivation associated with age differences in problem gambling in Japan. A total of 160 participants over 20 years of age who had gambled within the past six months were randomly recruited from web monitors. In this study, the Japanese version of the modified Gambling Motivation Scale (J-MGMS) was used which comprises six systematic factors: intellectual challenge, excitement, socialization (coping and sociability), social recognition, monetary gain, and amotivation. The Japanese version of the South Oaks Gambling Screening (J-SOGS) was used to assess participants’ gambling-related problems. Demographic data, such as gambling frequency, were solicited. Using linear regression analysis, amotivation in all participants, social recognition in early adults (under 30), and amotivation in late adults (30 or over) were associated with J-SOGS scores (adjusted R2 = 0.170, 0.290, 0.156). Among late adults, social recognition was nearly significant, although negative (*p* = 0.0503). 1) Self-determinant (autonomous) motivations such as excitement and socialization do not contribute to the progression of problem gambling. 2) Two non-self-determinant (non-autonomous) motivations, social recognition in early adults and amotivation in late adults, are predictors of problem gambling. 3) Social recognition is a dichotomic and paradoxical motivation in the progress of problem gambling according to age.

## Introduction

Gambling is illegal in Japan. However, gambling opportunities are abundant and more than half Japanese participate, because certain gambling industries are allowed as “amusement” or “public goods”. The former is an electronic gambling machine (EGM) called ‘pachinko and pachislot’, which is the most popular gambling form in Japan. It is not classified as gambling due to the prize exchange. The latter is a public-management gambling, namely state-regulated betting, such as horse racing, boat racing, and lotteries, etc. Thus, the social status of gambling in Japan is ambiguous.

Approximately 2.2% of the Japanese population has been estimated to have gambling problems (South Oaks Gambling Screen, SOGS 5+) (Ministry of Health, Labor & Welfare Japan, 2022). Gambling motivation has been found to play a dominant role in the progression of problem gambling (Blaszczynski et al., 2008; Daoud, 2017; Sundqvist et al., 2016). For successful interventions, it is important to reveal the nature of motivations associated with problem gambling (Carruthers et al., 2006).

In this paper, the term ‘‘problem gambling,” which is less specific than ‘‘gambling disorder’’ or “pathological gambling,” was adopted to emphasize a dimensional approach such as motivation-matched intervention, as opposed to a categorical approach (Shinaprayoon et al., 2017). Therefore, this study discussed “problem gambling” without examining whether individuals satisfied specific criteria.

Previous research has identified motivations that could lead to problem gambling using various gambling motivation scales such as the four-factor Gambling Motives Questionnaire-Financial (GMQ-F) scale (Dechant, 2014). The GMQ-F scale factors are negative reinforcement (to alleviate negative emotions), positive reinforcement (to induce emotions), social connections (to build relationships with others), and economic factors (to gain more money). Some researchers maintain that three motivations; negative reinforcement (escape/coping), positive reinforcement (enhancement/excitement), and economic factors (money motives) are associated with problem gambling (Barrada et al., 2019; Francis et al., 2015; Hearn et al., 2021; Wardle et al., 2011). Specifically, escape motives often lead to risky behavior in women, whereas enhancement motives often lead to risky behavior in men (Sundqvist et al., 2016).

There are other important motivations in addition to the above four. Some studies have revealed that problem gamblers play for challenges, control beliefs (competent), social interaction (sociality or to impress others), or apathy (without a purpose or reason, that is, amotivation) (Griffiths, 1990; Wood & Griffiths, 2007). Therefore, scales with four factors are insufficient for systematically assessing the association between gambling motivation and problem gambling.

The Modified Gambling Motivation Scale (MGMS), which was revised from the Gambling Motivation Scale (GMS) based on learning theory, has more factors and items than subsequent scales (Chantal et al., 1994; Karli, 2008; Komoto et al., 2022; Shinaprayoon et al., 2017; Wu & Tang, 2011). The GMS has a seven-factor structure with a seven-point Likert scale comprising: knowledge, accomplishment (to feel competent), excitement, socialization, social recognition (to feel important and increase self-esteem), monetary gain, and amotivation. In the GMS, social interaction and negative reinforcement (coping and relaxation) were combined into “socialization.” In contrast, the MGMS consists of six factors and has a new factor, namely “intellectual challenge,” instead of knowledge and accomplishment.

Studies using the GMS and MGMS have revealed that amotivation, accomplishment, and social recognition are associated with problem gambling (Chantal et al., 1994; Komoto et al., 2022; Marmurek & Cooper, 2023; Shinaprayoon et al., 2017; Wu & Tang, 2011).

The Japanese version of the MGMS (J-MGMS) with a dichotomous scale not only has the original six-factor structure but also a new four-factor structure. The four-factor structure is useful for motivation-matched interventions to discover adequate substitute behaviors (Komoto et al., 2022). In a previous study that used the J-MGMS with a four-factor structure, only amotivation was significantly associated with gambling problems in a linear regression analysis (Komoto et al., 2022). However, the J-MGMS with six factors was not analyzed in this previous study.

Generally, gambling motivation is considered to have different characteristics, depending on age. First, in adolescents and early adults, self-identity is often unstable because of negative self-image. Therefore, they are more ambivalent than older gamblers (Komoto et al., 2017) and ideal self-images brought about by winning are used to raise feelings of self-value. Namely, younger gamblers tend to gamble for winning money, socialization, accomplishment, and the “buzz’’ (i.e. impression made to others) (Felsher et al., 2004). Compared with younger gamblers, older gamblers tend to have more free time and disposable income for gambling (McNeilly & Burke, 2002). Therefore, their dominant motivation is to use gambling as a pastime. Furthermore, at their age, they may experience declines in social communication, separations from spouses or friends, tendencies to withdraw socially, and chronic disease, which could attract them to gambling as a momentary time killer from the empty hours ahead (McNeilly & Burke, 2000). Gambling is also a convenient pastime for busy middle-aged workers. Consequently, the gambling motivations of older adults may be simpler and more uncomplicated than that of younger people. The difference in gambling motivation between early and late adults is evidently not negligible. Therefore, differences between early and late adults must be analyzed to assess the association between gambling motivation and problem gambling.

### Purpose

This study aimed to examine the role of motivation in problem gambling in Japan. Additionally, the differences between early and late adults concerning gambling motivations and their association with problem gambling were examined. These insights can guide the development of tailored interventions for problem gambling.

### Hypothesis

First, younger gamblers are more ambivalent than older gamblers and are expected to be more motivated to gamble than older gamblers. Second, young gamblers with problem gambling expect to increase their self-image, namely, social recognition. Third, older gamblers with problem gambling are expected to gamble as a pastime, namely, through amotivation. Finally, Japanese gamblers with self-determinant (autonomous) motivations, such as excitement and socialization, are not expected to progress to gambling problems.

## Methods

### Participants

Candidates were recruited in July 2019 through the online recruitment website ASMARQ Co. Ltd. and provided their written informed consent to participate. First, a survey was completed by 1,145 individuals who had gambled within the past six months, were residents of the Tokyo metropolitan area, and were aged 20 years or older. Next, 160 participants were randomly selected out of the 1,145 candidates, ensuring approximately equal numbers of male and female respondents for each age group (from 20’ to 60’ years), namely, 32 participants (16 males and 16 females) for each group.

### Measures

#### Modified Gambling Motivation Scale Japanese Version (J-MGMS)

We used a dichotomous J-MGMS format with a six-factor structure (Komoto et al., 2022). Only the dichotomous format of the J-MGMS had good factor validity (RMSEA = 0.063; SRMR = 0.072).

The six factor-structure of the J-MGMS consisted of “intellectual challenge” (e.g., ‘‘I enjoy learning new strategies’’ and “I feel competent when I gamble”), excitement (e.g., ‘‘It is exciting to gamble’’), socialization (e.g., ‘‘It is the best way to relax’’), social recognition (e.g., ‘‘It makes me feel important’’), monetary gain (e.g., ‘‘I play for money’’) and amotivation (e.g., ‘‘I play for money, but I sometimes wonder what I get out of gambling’’).

The internal consistencies (α value) of the six-factor structure of J-MGMS were excellent; total scores (0.960), intellectual challenge (0.893), excitement (0.865), socialization (0.861), social recognition (0.880), monetary gain (0.892) and amotivation (0.888). According to the self-determined theory (SDT), which is the fundamental theory for MGMS, these six factors are ordered from the most autonomous (self-determined or intrinsic) motivations to the least autonomous (non-self-determined or extrinsic) motivations (Chantal et al., 1994). The most autonomous motivation is an intellectual challenge, followed successively by excitement, socialization, social recognition, and monetary gain. The least autonomous motivation is amotivation.

#### Scoring System for the J-MGMS

Participants completed 28 items (four or eight items per factor) using a dichotomous scale (1 = no, 2 = yes). Higher scores indicate a more intense motivation to gamble for a specific reason or in general. Each subscale score ranges from 4 to 8, although the intellectual challenge score ranges from 8 to 16. This scoring allows researchers to assess the systematic psychological structure of the motivation to gamble.

#### The South Oaks Gambling Screening Japanese Version (J-SOGS)

The modified Japanese version of the SOGS (J-SOGS) (Kido & Shimazaki, 2007) was used to assess participants’ gambling-related problems in the previous year, because this study targeted not a clinical sample, but a population sample. The scale was developed based on old DSM-III criteria for pathological gambling. Participants rated 20 items on a dichotomous scale (0 = no, 1 = yes) to assess their risk of gambling problems resulting in a score between 0 and 20.

#### Demographic Questionnaire

The demographic questionnaire measured sex (male or not), age (early adults: age 20–29 or late adults: 30–69 years), present marital status (married or not), present occupation (working or not), the most dominant gambling type (pachinko and pachi slot or others), and gambling frequency (less than once a week or at least once a week). In Japan, Pachinko and Pachi-slots are the most popular forms of gambling. They are categorized as non-strategic gambling and are played individually in parlors using arcade game-like machines.

#### Procedure

The recruitment and selection of participants were performed according to ASMARQ’s ethical rules. All participants were provided with information about the study, and informed consent was obtained. This study was approved by the Ethical Review Board for Research of Yoshino Hospital. The study was performed in accordance with the ethical standards as laid down in the 1964 Declaration of Helsinki and its later amendments or comparable ethical standards.

#### Statistical Analysis

Data entry and statistical analysis were performed using Microsoft Excel® 2021 MSO. Pearson correlation analysis was used to assess the correlations between the J-MGMS and the six-factor scores, demographic variables, and J-SOGS scores. Each correlation level was judged by correlation coefficient values as Weak (-0.3), Moderate (0.4–0.6), or Strong (0.7-) (Akoglu, 2018).

To evaluate motivational predictors independently associated with problem gambling, a multivariate analysis was performed using a linear regression model. In this model, the dependent variable was the total J-SOGS score. The explanatory variable was the average score of the six factors of the J-MGMS.

The differences between early adults and late adults were investigated by a χ2-test or t-test. Like the analysis of all participants, Pearson’s correlation analysis and multivariate regression analysis using a linear regression model were performed for each group.

Multicollinearity leads to incorrect regression results. Therefore, variance inflation factor (VIF) was used as a diagnostic tool for multicollinearity. If VIF is greater than 10, the specified factor must be removed because of multicollinearity (Daoud, 2017). Statistical significance was set at *p* < 0.05.

## Results

### Demographic Description

The sample consisted of 160 participants (50% men and 50% women). Of these early adults (under 30) comprised 20.0%, participants who were married comprised 66.8%, those with occupations comprised 91.9%, participants who dominantly played Pachinko & Pachi-slot comprised 53.1%, and frequent players (at least once a week) comprised 45.0%.

The only significant demographic difference between the early and late adults was marital status (*p* = 0.002). Moreover, early adults had significantly more gambling problems than late adults (*p* < 0.001) (Table 1).

**Table 1:** Demographic description

Variables	Total (*N* = 160)	Total (*N* = 160)	Early adult group (*N* = 32)	Late adult group (*N* = 128)	
	Frequency (%)	Number (%)	Number (%)	Number (%)	χ2-test
Male	80 (50.0)	80 (50.0)	16 (50.0)	64 (50.0)	
Early adult (20–29)	64 (40.0)	32 (20.0)			
Married	98 (61.3)	107 (66.8)	12 (37,5)	87 (68.0)	*
Employed	98 (61.3)	147 (91.9)	30 (93.8)	117 (91.5)	
Pachinko & Pachi-slot	85 (53.1)	85 (53.1)	14 (43.8)	71 (55.5)	
Gambling Frequency (at least once a week)	88 (55.0)	72 (45.0)	15 (46.9)	57 (44.5)	
					t-test
SOGS score: average (SD)		5.43(4.25)	8.16(4.30)	4.74 (3.97)	*
*p < 0.05					

SOGS, South Oaks Gambling Screening

### Motivation Description

Excitement is the most common motivation for gambling. The ratio of the number of yes answers to the excitement factor for all participants (average % yes for excitement) was 65.0%. The average percentage of yes responses for each factor was as follows: 48.3% (amotivation), 41.5% (monetary), 35.3% (intellectual challenge), and 34.2% (socialization). Social recognition was the least common motivation for participating (18.2%). Moreover, early adults had significantly more gambling motivation than late adults (*p* < 0.001–0.013) (Table 2).

**Table 2:** Japanese version of the modified gambling motivation scale description

	Total (*N* = 160)	Total (*N* = 160)		Early adult group (*N* = 32)	Late adult group (*N* = 128)	
Factor	Frequency (%)	Average %yes		Average %yes	Average %yes	t-test
Intellectual challenge	80 (50.0)	35.3		**47.7**	32	*
Social recognition	64 (40.0)	18.2		**39.1**	13.9	*
Excitement	98 (61.3)	65		71.1	61.6	
Socialization	98 (61.3)	34.2		44.5	31.5	
Monetary gain	85 (53.1)	41.5		**58.6**	37.3	*
Amotivation	88 (55.0)	48.3		**65.6**	43.9	*
Average %yes: percentage of yes-answer in each factor					*p < 0.05	

### Correlation Analysis

In early adults, gambling frequency (the number of frequent gamblers) and all six factors of the J-MGMS had a significant positive correlation with the J-SOGS (*r* = 0.317–0.418). Additionally, amotivation was moderate (Table 3).In early adults, two factors of the J-MGMS, intellectual challenge and social recognition, had moderately significant correlations with J-SOGS scores (*r* = 0.434–0.559) (Table 4). In late adult pachinko and pachi slot players, gambling frequency (frequent gambler), and almost all factors of the J-MGMS, except social recognition, had a significant positive correlation with the J-SOGS (*r* = 0.193–0.403). However, only amotivation was moderate (Table 5).

**Table 3:** Association between demographic variables, motivation factors and South Oaks Gambling Screening score in total participants

	Early adult	Male	Married	Employed	Pachinko & pachi-slot	Gambling frequency	Intellectual challenge	Social Recognition	Excitement	Socialization	Monetary gain	Amotivation
SOGS score	0.322	0.115	0.0726	0.0945	0.132	0.25	0.354	0.317	0.223	0.323	0.322	**0.418**
	*					*	*	*	*	*	*	*

*p < 0.05

Bold: moderate association

SOGS, South Oaks Gambling Screening

**Table 4:** Association between demographic variables, motivation factors, and South Oaks gambling screening score in early adult group

	Male	Married	Employed	Pachinko & pachi-slot	Gambling frequency	Intellectual challenge	Social Recognition	Excitement	Socialization	Monetary gain	Amotivation
SOGS score	0.288	0.185	-0.143	-0.152	0.261	**0.434**	**0.559**	0.189	0.336	0.276	0.272
						*	*				

*p < 0.05

Bold: moderate association

SOGS, South Oaks Gambling Screening

**Table 5:** Association between demographic variables, motivation factors, and South Oaks gambling screening score in late adult group

	Male	Married	Employed	Pachinko & pachi-slot	Gambling frequency	Intellectual challenge	Social Recognition	Excitement	Socialization	Monetary gain	Amotivation
SOGS score	0.076	0.163	0.142	0.259	0.257	0.277	0.124	0.193	0.282	0.268	**0.404**
				*	*	*		*	*	*	*

*p < 0.05

Bold: moderate association

### Multivariate Analysis

Linear regression analysis revealed that only amotivation was significantly associated with SOGS scores in all participants. This variable accounted for 17.0% of J-SOGS scores (adjusted R2 = 0.170) (Table 6).In early adults, only social recognition was significantly associated with the SOGS scores. This variable accounted for 29.0% of J-SOGS scores (adjusted R2 = 0.290) (Table 7).In late adulthood, only amotivation was significantly associated with J-SOGS scores. This variable accounted for 15.6% of J-SOGS scores (adjusted R2 = 0.156). Social recognition was nearly significant, although negative (*p* = 0.0503; standardized partial regression coefficient=-0.220) (Table 8). None of the VIF values were greater than 10; therefore, no variables needed to be removed because of strong multicollinearity.

**Table 6:** Result of the linear regression analysis with South Oaks gambling screening scores pertaining to the six-factor Japanese version of the modified Gambling motivation scale in total participants

No.	Variables	Partial regression Coefficients	standard error	t-value	*p*-value		Standardized partial regression coefficient	Variance inflation factor
1	Intellectual challenge	0.1203	0.1890958	0.63621	0.52559		0.06894	2.3317
2	Social recognition	0.13457	0.3542277	0.37991	0.70454		0.03932	2.127
3	Excitement	-0.14864	0.3014484	0.49311	0.62264		-0.044355	1.6062
4	Socialization	0.43871	0.3059176	1.43411	0.15358		0.14474	2.0224
5	Monetary gain	0.26886	0.2796519	0.96143	0.33785		0.094622	1.9229
6	Amotivation	0.80556	0.2235131	3.60412	**0.00042**	*	0.29905	1.3669
7	Intercept	-16.6761	2.0149418	8.27622	5.9E-14			

*p < 0.05 (adjusted R^2^ = 0.170)

**Table 7:** Result of the linear regression analysis with South Oaks Gambling Screening scores pertaining to the six-factor Japanese version of the modified gambling motivation scale in early adult participants

No.	Variables	Partial regression Coefficients	Standard error	T-value	*P*-value		Standardized partial regression coefficient	Variance inflation factor
1	Intellectual challenge	0.34504	0.5661281	0.60948	0.5477031		0.19718	4.2461
2	Social recognition	2.46634	0.9121814	2.70379	**0.0121511**	*	0.84943	4.0039
3	Excitement	1.15249	0.9267133	1.24364	0.225168		0.32754	2.8139
4	Socialization	-1.565252	1.0704387	1.46225	0.1561303		-0.529444	5.3182
5	Monetary gain	-0.510558	0.8457404	0.60368	0.5514944		-0.1527489	2.5972
6	Amotivation	-0.189733	0.6618275	0.28668	0.7767201		-0.067999	2.2823
7	Intercept	-20.53962	4.0876778	5.02476	3.496E-05			

*p < 0.05 (adjusted R^2^ = 0.290)

**Table 8:** Result of the linear regression analysis with South Oaks gambling screening scores pertaining to the six-factor Japanese version of the modified gambling motivation scale in late adult participants

No.	Variables	Partial regression Coefficients	Standard error	T-value	*P*-value		Standardized partial regression coefficient	Variance inflation factor
1	Intellectual challenge	0.186843	0.1922307	0.97198	0.3330014		0.111785	2.0506
2	Social recognition	-0.80012	0.4046912	1.97711	0.0503025		-0.220053	1.9264
3	Excitement	-0.12281	0.3088361	0.39766	0.6915815		-0.039295	1.5185
4	Socialization	0.56149	0.3107065	1.80715	0.073223		0.194488	1.8011
5	Monetary gain	0.36849	0.292943	1.25792	0.2108438		0.139185	1.9031
6	Amotivation	0.85072	0.229975	3.6992	**0.0003266**	*	0.331024	1.2452
7	Intercept	-12.1077	2.3908053	5.0643	1.486E-06			

*p < 0.05 (adjusted R^2^ = 0.156)

## Discussion

This study used the J-MGMS to investigate gambling motivations and their association with problem gambling among Japanese gamblers. Additionally, differences in gambling motivation between early and late adults were investigated.

Confirming previous studies, early adults are more often engaged in problem gambling than late adults are (Buth et al., 2017; Hing & Russell, 2020). In addition, unstable self-identity and delayed discounting and impulsivity (the tendency to act rashly) were identified in young, disordered gamblers (Steward et al., 2017). Furthermore, both positive and negative urgency is a mediator of severity levels in young problem gamblers (Blaszczynski & Nower, 2002). Generally, greater gambling motivations lead to gambling problems (Clarke, 2008; Shinaprayoon et al., 2017). Additionally, this study revealed that early adults were more motivated to gamble than late adults.

In this study, excitement (positive enhancement) was the most common motivation for gambling, as has been shown in previous studies (Auer & Griffiths, 2023). However, among the six motivation factors, excitement was the least associated with problem gambling. Additionally, excitement was far from problem gambling in the linear regression analysis of all participants (standardized partial regression coefficient: -0.0444). Therefore, excitement may be a key motivation for preventing problem gambling among Japanese gamblers.

Contrary to this Japanese finding, most American-European studies revealed that intrinsic motivations such as excitement and coping were associated with problem gambling (Shinaprayoon et al., 2017; Wu & Tang, 2011). Previous studies in East Asia, including this study, have revealed that amotivation is an independent predictor of problem gambling (Komoto et al., 2022; Wu & Tang, 2011). This difference may be caused by cultural differences concerning collectivism versus individualism; namely, East Asians, including Japanese gamblers, are easily influenced by surrounding social relationships and forget their individual, original reasons for gambling (i.e., amotivation occurs) (Komoto et al., 2022).

Another noteworthy finding of this study is that the association between amotivation and problem gambling is limited to late adulthood. Older adults often have decreased self-control owing to diminished executive function, which is a possible contributor to pathological gambling (von Hippel et al., 2009). This opaque mood leads to aimless gambling – that is, gambling with amotivation. Therefore, for older adults, gambling with amotivation may often lead to problematic gambling behaviors. However, social recognition is not a problem motivation, but an anti-problem motivation in older adults. This motivation may prevent a fall into gambling through amotivation by providing older adults with a modest aim.

In contrast, amotivation was found not to be an independent predictor of problem gambling in early adults. In other words, amotivation does not play a dominant role in the progression of problem gambling. In general, early adults are more ambivalent or uncertain than late adults (Komoto et al., 2017) but this study found that early adults had more motivation than late adults. Accordingly, equivocal amotivation does not always lead to problem gambling in early adults who regularly gamble. For them, pastimes with amotivation may be a popular motivation for habitual behaviors, not limited to gambling.

In early adulthood, intellectual challenges and social recognition are moderately associated with problem gambling. Social recognition was found in this study to be an independent predictor of problem gambling. Therefore, intellectual challenges may lead to problem gambling through social recognition. In a study of university students, pathological gamblers gamble, not for the needs gained from the act of gambling itself, but for extrinsic reasons (Felsher et al., 2004). They gamble to satisfy their ego needs or increase their self-esteem (social recognition). In this young sample, intrinsic motivations, such as stimulation (excitement), had a weaker association with pathological gambling than extrinsic motivations. Another study revealed that problem gamblers were motivated to gamble for self-esteem, such as feeling important in the eyes of others (social recognition) (Walters, 1994). Individuals with a weak sense of self-worth may be focused on the momentary boost in self-esteem brought about by a win (Ryan & Deci, 2000). Therefore, motivation for social recognition in early adulthood often becomes more compulsive than in late adulthood because of a negative self-image. For these individuals, chasing losses may be equivalent to increasing self-esteem.

From the above, in this study three important findings were revealed:

1. Self-determined (autonomous) motivations, such as excitement (positive reinforcement) and socialization (negative reinforcement), do not contribute to progress in problem gambling.
2. Two non-self-determining (non-autonomous) motivations are important predictors of problem gambling: social recognition in early adulthood and amotivation in late adulthood.
3. Social recognition is a dichotomic and paradoxical motivation in the progress of gambling problems.

These results should be understood in the context of the limitations of this study. First, this study employed self-reported data with some biases. Second, the data excluded individuals age 70 or older. Retired people in their 70s may have different motivations for gambling, such as battling boredom and loneliness. (Guillou et al., 2019). Third, the SOGS does not rate pathological gambling. Therefore, the characteristic motivations for pathological gamblers, such as self-medication, were not identified in this study. Forth, the data were analyzed using cross-sectional methods, which made it difficult to conclude a causal relationship between motivation and other variables. Future longitudinal research should investigate the causality between gambling motivation and problem gambling. Fifth, other potential psychosocial factors affect gambling motivations such as emotion regulation (Marchica et al., 2020). Future studies should investigate these additional factors.

Despite these limitations, this study revealed that non-self-determined motivations, such as social recognition in early adults and amotivation in late adults, more strongly influenced the progression of gambling problems than self-determined motivations, such as excitement and socialization, which are predictive motivations for gambling problems in Western cultures. By identifying the motivations associated with the progression of problem gambling at different ages, this study has significant implications for the development of tailored interventions to prevent problem gambling in Japan. Clinicians should, for example, take cognizance of the motivational differences between Western and Eastern cultures and between gamblers in early and late adulthood.

## Data Availability

The datasets generated during and/or analysed during the current study are available from the corresponding author on reasonable request.

## References

[CR1] Akoglu, H. (2018). User’s guide to correlation coefficients. *Turkish Journal of Emergency Medicine*, *18*(3), 91–93. 10.1016/j.tjem.2018.08.001 30191186 10.1016/j.tjem.2018.08.001 PMC6107969

[CR2] Auer, M., & Griffiths, M. D. (2023). Reasons for gambling and problem gambling among Norwegian horse bettors: A real-world study utilizing combining survey data and behavioral player data. *International Journal of Mental Health and Addiction*, *21*(2), 740–755. 10.1007/s11469-020-00442-6

[CR3] Barrada, J. R., Navas, J. F., Ruiz de Lara, C. M., Billieux, J., Devos, G., & Perales, J. C. (2019). Reconsidering the roots, structure, and implications of gambling motives: An integrative approach. *PLOS ONE*, *14*(2), e0212695. 10.1371/journal.pone.0212695 30794642 10.1371/journal.pone.0212695 PMC6386301

[CR4] Blaszczynski, A., & Nower, L. (2002). A pathways model of problem and pathological gambling. *Addiction*, *97*(5), 487–499. 10.1046/j.1360-0443.2002.00015.x 12033650 10.1046/j.1360-0443.2002.00015.x

[CR5] Blaszczynski, A., Walker, M., Sharpe, L., & Nower, L. I. A. (2008). Withdrawal and tolerance during in problem gambling. *International Gambling Studies*, *8*(2), 179–192. 10.1080/14459790802140007

[CR6] Buth, S., Wurst, F. M., Thon, N., Lahusen, H., & Kalke, J. (2017). Comparative analysis of potential risk factors for at-risk gambling, problem gambling and gambling disorder among current gamblers–results of the Austrian representative survey 2015. *Frontiers in Psychology*, *8*, 2188. 10.3389/fpsyg.2017.02188 29312056 10.3389/fpsyg.2017.02188 PMC5735080

[CR7] Carruthers, C., Platz, L., & Busser, J. (2006). Gambling motivation of individuals who gamble pathologically. *Therapeutic Recreation Journal*, *40*(3), 165.

[CR8] Chantal, Y., Vallerand, R. J., & Vallères, E. F. (1994). Construction Et validation de l’echelle de motivation relative aux jeux de hasard et d’argent [Assessing motivation to gamble: On the development and validation of the Gambling Motivation Scale]. *Loisir et Société / Society and Leisure*, *17*(1), 189–212. 10.1080/07053436.1994.10715471

[CR9] Clarke, D. (2008). Older adults’ gambling motivation and problem gambling: A comparative study. *Journal of Gambling Studies*, *24*(2), 175–192. 10.1007/s10899-008-9090-z 18273695 10.1007/s10899-008-9090-z

[CR10] Daoud, J. I. (2017). Multicollinearity and regression analysis. *Journal of Physics: Conference Series*. IOP Publishing, *949*(1), 012009. 10.1088/1742-6596/949/1/012009

[CR11] Dechant, K. (2014). Show me the money: Incorporating financial motives into the gambling motives questionnaire. *Journal of Gambling Studies*, *30*(4), 949–965. 10.1007/s10899-013-9386-5 23740347 10.1007/s10899-013-9386-5

[CR12] Felsher, J. R., Derevensky, J. L., & Gupta, R. (2004). Lottery participation by youth with gambling problems: Are lottery tickets a gateway to other gambling venues? *International Gambling Studies*, *4*(2), 109–125. 10.1080/14459790412331296956

[CR13] Francis, K. L., Dowling, N. A., Jackson, A. C., Christensen, D. R., & Wardle, H. (2015). Gambling motives: Application of the reasons for gambling questionnaire in an Australian population survey. *Journal of Gambling Studies*, *31*(3), 807–823. 10.1007/s10899-014-9458-1 24705633 10.1007/s10899-014-9458-1

[CR14] Griffiths, M. D. (1990). The acquisition, development, and maintenance of fruit machine gambling in adolescents. *Journal of Gambling Studies*, *6*(3), 193–204. 10.1007/BF01014578 24242894 10.1007/BF01014578

[CR15] Guillou, L. M., Cholet, J., Grall, B. M., Lalande, S., & Le, R. J. Y. (2019). Determinants of gambling disorders in elderly People—A systematic review. *Frontiers in Psychiatry*, *10*, 837. 10.3389/fpsyt.2019.00837/full. https://www.frontiersin.org/journals/psychiatry/articles/31824348 10.3389/fpsyt.2019.00837 PMC6886010

[CR16] Hearn, N. L., Ireland, J. L., Eslea, M., & Fisk, J. E. (2021). Exploring pathways to gambling: Proposing the integrated risk and protective factors model of gambling types. *Journal of Gambling Studies*, *37*(1), 1–26. 10.1007/s10899-020-09929-2 31965384 10.1007/s10899-020-09929-2 PMC7882557

[CR17] Hing, N., & Russell, A. M. T. (2020). Proximal and distal risk factors for gambling problems specifically associated with electronic gaming machines. *Journal of Gambling Studies*, *36*(1), 277–295. 10.1007/s10899-019-09867-8 31172326 10.1007/s10899-019-09867-8

[CR18] Karli, Ü. (2008). *The determination of motivational factors of sport gambling university students and their personality and psychological differences from non-gamblers*. http://etd.lib.metu.edu.tr/upload/3/12610087/index.pdf [Unpublished Doctoral Dissertation]. ODTÜ METU.

[CR19] Kido, M. & Shimazaki, T. (2007). Reliability and validity of the modified Japanese version of the South oaks Gambling screen (SOGS). *Shinrigaku Kenkyu (Japanese Version)* 77 6 547–552 10.4992/jjpsy.77.547 10.4992/jjpsy.77.547 17447464

[CR21] Komoto, Y., Shoun, A., Akiyama, K., Sakamoto, A., Sato, T., Nishimura, N., Shinohara, K., Ishida, H., & Makino, N. (2017). Development and validation of the Pachinko/Pachi-Slot playing ambivalence scale. *Asian Journal of Gambling Issues and Public Health*, *7*(1), 3. 10.1186/s40405-017-0023-6 28573085 10.1186/s40405-017-0023-6 PMC5429383

[CR20] Komoto, Y., Kaneko, M., & Nobayashi, K. (2022). *Development and evaluation of a modified gambling motivation scale* (Japanese version). *Journal of Gambling Issues*, (50).

[CR22] Marchica, L. A., Keough, M. T., Montreuil, T. C., & Derevensky, J. L. (2020). Emotion regulation interacts with gambling motives to predict problem gambling among emerging adults. *Addictive Behaviors*, *106*, 106378. 10.1016/j.addbeh.2020.106378 32203700 10.1016/j.addbeh.2020.106378

[CR23] Marmurek, H. H., & Cooper, A. (2023). Dichotomous and weighted scoring of the problem gambling severity index converge on predictors of problem gambling. *International Journal of Mental Health and Addiction*, 1–14.

[CR24] McNeilly, D. P., & Burke, W. J. (2000). Late life gambling: The attitudes and behaviors of older adults. *Journal of Gambling Studies*, *16*(4), 393–415. 10.1023/a:1009432223369 14634305 10.1023/a:1009432223369

[CR25] McNeilly, D. P., & Burke, W. J. (2002). Disposable time and disposable income: Problem casino gambling behavior in older adults. *Journal of Clinical Geropsychology*, *8*(2), 75–85. 10.1023/A:1014679507988

[CR26] Ministry of Health, Labor and Welfare Japan (2022/3/25). Basic counterplan for gambling problem. https://www.kantei.go.jp/jp/singi/gambletou_izonsho/pdf/kihon_keikaku_honbun_20220325.pdf

[CR27] Ryan, R. M., & Deci, E. L. (2000). Self-determination theory and the facilitation of intrinsic motivation, social development, and well-being. *American Psychologist*, *55*(1), 68–78. 10.1037/0003-066X.55.1.68 11392867 10.1037//0003-066x.55.1.68

[CR28] Shinaprayoon, T., Carter, N. T., & Goodie, A. S. (2017). The modified gambling motivation scale: Confirmatory factor analysis and links with problem gambling. *Journal of Gambling Issues*, *37*. 10.4309/jgi.2018.37.5

[CR29] Steward, T., Mestre-Bach, G., Fernández-Aranda, F., Granero, R., Perales, J. C., Navas, J. F., Soriano-Mas, C., Baño, M., Fernández-Formoso, J. A., Martín-Romera, V., Menchón, J. M., & Jiménez-Murcia, S. (2017). Delay discounting and impulsivity traits in young and older gambling disorder patients. *Addictive Behaviors*, *71*, 96–103. 10.1016/j.addbeh.2017.03.001 28288442 10.1016/j.addbeh.2017.03.001

[CR30] Sundqvist, K., Jonsson, J., & Wennberg, P. (2016). Gambling motives in a representative Swedish sample of risk gamblers. *Journal of Gambling Studies*, *32*(4), 1231–1241. 10.1007/s10899-016-9607-9 27038813 10.1007/s10899-016-9607-9

[CR31] von Hippel, W., Ng, L., Abbot, L., Caldwell, S., Gill, G., & Powell, K. (2009). Executive functioning and gambling: Performance on the trail making test is associated with gambling problems in older adult gamblers. *Neuropsychology Development and Cognition Section B*, *16*(6), 654–670. 10.1080/13825580902871018 10.1080/13825580902871018 19484614

[CR32] Walters, G. D. (1994). The gambling lifestyle: I. Theory. *Journal of Gambling Studies*, *10*(2), 159–182. 10.1007/BF02109938 24234840 10.1007/BF02109938

[CR33] Wardle, H., Moody, A., Spence, S., Orford, J., Volberg, R., Jotangia, D., & Dobbie, F. (2011). British gambling prevalence survey 2010 prepared for. https://assets.publishing.service.gov.uk/government/uploads/system/uploads/attachment_data/file/243515/9780108509636.pdf. *The Gambling Commission*.

[CR34] Wood, R. T., & Griffiths, M. D. (2007). A qualitative investigation of problem gambling as an escape-based coping strategy. *Psychology and Psychotherapy*, *80*(1), 107–125. 10.1348/147608306X107881 17346384 10.1348/147608306X107881

[CR35] Wu, A. M., & Tang, C. S. K. (2011). Validation of the Chinese version of the gambling motivation scale (C-GMS). *Journal of Gambling Studies*, *27*(4), 709–724. 10.1007/s10899-010-9234-9 21191635 10.1007/s10899-010-9234-9

